# Translations from Achives Generales De Medicine, for December, 1850

**Published:** 1851-03

**Authors:** 


Art. II. — Translations from *Archives Generales de Medicine*, for December, 1850. Translated by Wm. H. Cobb, M.D., of Louisville, Ky.

M. Fuster, a Professor in the Medical Faculty of Montpellier, sent to the Academy of Medicine, on the 19th of November, a communication upon arsenical medication in intermittent fever. This communication resolves itself into the following propositions:

1st. Inveterate cases of intermittent fever, even when proving rebellious to the use of quinine, yield readly to the exhibition of arsenic.

2d. If the arsenic prove inefficient in producing a cure, quinine invariably proves remedial.

We commence, in this variety of fevers, with five centigrammes of the arsenic acid, taken three times during the day, and increase the dose according to the indications to nine or ten centigrammes. The best formula for its administration, whether it be exhibited by the mouth or given in the form of enema, is to incorporate it with twenty times its bulk of sugar of milk as soon as the disease has been subdued, the dose is to be diminished in the same ratio that it was increased, until the initial dose be attained. No bad effects attend its use; the medicine is generally well borne by the sick. In the regions of country south of Montpellier, emetics, given at the commencement and in the course of the treatment, favor, maintain or reëstablish the tolerance of the acid.

No special regimen is required, except on those days that the emetics are administered. The visceral engorgements attendant upon the fever are never resolved as speedily as the disease itself, they only diminish, and finally disappear, after the general health has been permanently reëstablished.

Dr. Aran, physician to the central office of the Hospitals, presented a paper upon "the local use of Anæsthetic Agents," and more especially upon the relative value of anæsthetic agents with a view to this medication. The following are the conclusions at which he arrived:

1st. All volatile substances which we recognize as possessing general anæsthetic properties (the different varieties of ether, chloroform, Dutch oil, or chloruret of olefiant gas, aldehyd, benzoin) also possess local anæsthetic powers when applied endermically.

2d. The intensity of their local anæsthetic power is not in proportion to their general effect, but rather in an inverse ratio to it. This is the explanation of the fact that, when considered with a view to its local anæsthesia, Dutch oil exceeds all the aforementioned substances, that chloroform is superior to the ethers, and that acetic ether surpasses the other kinds of ether.

3d. Of the different anæsthetic agents, some, as the ethers, aldehyd and benzoin, can be applied to the skin, even in an undiluted form, without producing any irritation; while others, on the contrary, possess an acrimony which render them more or less irritating, chloroform, even when of the purest form, is of this class; when applied in small quantity, it causes a slight sensation of warmth, which gradually increases to a sensation of extreme heat, and even determines vesication if the contact be prolonged, or the quantity of chloroform poured upon the skin be at all considerable. This irritating action is developed in a more energetic manner if the evaporation of the chloroform from the surface where it was applied, be allowed to proceed freely. The Dutch oil also possesses irritating properties but to a much less extent.

4th. As before stated, the best of all the anæsthetics, with a view to their local application, is the Dutch oil, or chloruret of olefiant gas; its anæsthetic action continues longer even when employed in small quantities, from fifteen to twenty drops, upon the painful surface; it produces but a very insignificant amount of cutaneous irritation; whereas chloroform induces heat and pain. The former has an ethereal odor, slight and rather agreeable than otherwise; while on the other hand, the ethers, aldehyd and benzoin, possess a pungent odor, which is illy borne by the sick; aldehyd and benzoin, in particular, have a very disagreeable smell when concentrated. The only objection to the introduction of the Dutch oil into general use is that in the present state of its manufacture, its price is very dear; but the opinion seems to be generally entertained, that in a short time its price will be reduced to the same as that of chloroform.

5th. In order to obtain sufficient local anæsthesia, it is not at all necessary to employ a large quantity; with from 15 to 20 gtts., at the farthest, of the Dutch oil poured upon the part affected, and covered with a wet compress and a piece of oilcloth, we can allay the pain, in the greater number of cases indicating the use of anæsthetic agents.

At the same session of the Academy of Medicine, a memoir was read by Dr. Sestier, upon "Intra-laryngeal œdema, and œdema affecting the back part of the mouth, supervening in cases of œdematous laryngitis." The author has collected 41 cases of œdematous laryngitis with œdema of the interior of the larynx; the œdema was sometimes general, (in 23 cases), sometimes partial, (in 18 cases). But whether it be general or partial, it almost invariably affects the vocal chords, causing a contraction of the glottis. This lesion is met with quite frequently in œdematous laryngitis, in about four-sevenths of the cases. It is important to consider this affection, because it tends to obscure the diagnosis of œdematous laryngitis, by substituting for the characteristic contrast of expiration and inspiration, a difficulty of breathing, because it aggravates the dangers already attendant upon the disease from the infiltration of the superior folds of the mucous membrane of this organ; and especially with a view to its treatment, as it renders the necessity for bronchotomy more necessary than when the superior folds are alone infiltrated.

Relative to the diagnosis of intra-laryngeal œdema, the author imposes the following conditions:

1st. Pay attention to the state of the patient preceding the attack, inasmuch as œdema within the larynx has only been clearly proved to exist when the patient was more or less gravely affected prior to the onset of the disease.

2d. Examine the etiological form of the disease, as intra-laryngeal œdema has never been observed when the affection of the larynx has been consequent upon inflammation of the throat, occurring in an individual previously enjoying good health, but very rarely in œdematious angina superadded to general inflammation, or in the case of a serous diathesis more or less advanced and indicated by anasarca or other serous effusion. The presence of laryngitis, whether it be acute, subacute or chronic, would lead us to suspect that the interior of the larynx is invaded by the œdema, as occurs in threesevenths of the cases. The supposition is rendered the more probable if the angina be connected with a serous diathesis; the proportion being three-fifths of the cases.

3d. Observe whether the contrast between the difficulty of inspiration and the facility of expiration exists or not; for in seven-twelfths of the cases in which this marked difference did not exist, still the œdema affected the interior of the larynx.

4th. Finally, inspect with care the back part of the mouth and assure yourself whether this region is infiltrated or not; for in twelve examinations of œdematous angina, with œdema extending more or less from the throat; intra-laryngeal effusion existed eleven times.

The œdema of the back of the mouth is sometimes general, sometimes partial; if it be partial, it may be confined to so small a space as to require a very minute and attentive examination to discover it. The uvula is most frequently affected; then come, in the order of their frequency, the soft palate, base of the tongue, pillars of the palate, sides of the cheeks, tonsils, glosso-epiglotic fold, pharynx, and lastly, the palatine arch. The infiltration of the back part of the mouth often presents the character of phlegmonous œdema; the infiltrated liquid is sometimes situated deeply in the submucous cellular tissue, sometimes almost beneath the epithelium; in which case the œdematous portion presents the appearance of a distended vesicle, vesicular polypus, or phlyctæna. Œdema at the posterior part of the mouth has been noticed in one-fourth of the cases. It is very frequent in œdematous angina consequent upon acute inflammation of the throat, but it appears more especially in the cases of angina occurring during the course of anasarca or general dropsy. It is important to consider this œdema, because it may enable us to prevent the subsequent invasion of œdematous laryngitis to discover it at its first onset by giving a special significance to the slight gutteral and laryngeal symptoms which so obscurely mark the first moments of the affection, to facilitate the diagnosis after its complete development, and finally to guide us in the choice of our therapeutic means. The author particularly recommends, among other direct means likely to prove successful, scarifications or incisions into the infiltrated parts. These scarifications, according to the author, may be very useful, if the laryngeal infiltration depend upon any obstacle to the venous circulation, having for its seat the heart or the course of the large cervical veins, or when dependent upon anasarca consecutive to scarlatina or miliary fever, upon a dropsical cachexy supervening upon repeated attacks of intermittent fever, upon scorbutic or cancerous affections; in the case of laryngeal œdema supervening immediately or after a short time upon wounds of the neck, with serous infiltration into the cellular tissue around the larynx. The chances of success are notably less if the laryngeal œdema be symptomatic of a very slight inflammation of the throat or larynx, or consecutive to a chronic laryngitis. The chances are almost nothing if the laryngeal inflammaton be owing to an acute and intense inflammation of the throat and larynx. At all times, it is probable that, even in this last form of the disease, scarifications would succeed, if resorted to in the first moments of the formation of the inflammatory œdema, because at that period we sometimes find merely a limpid serosity soon to be replaced by plastic lymph.

Dr. A. Mayer, of Belfort, addressed to the Academy of Medicine, November 26th, a memoir entitled, "The use of multiple scarification of the neck of the uterus, effected by means of a new instrument, in the treatment of leucorrhea symptomatic of uterine engorgement."—The author proposes to establish in this memoir,

1st. That there exists a variety of leucorrhea symptomatic of acute or chronic engorgement of the womb or of the neck alone.

2d. That this variety, rebellious under the usual modes of treatment, requires local sanguineous depletions.

3d. That leeches applied at a distance from the neck are inefficient, and that their application to the neck itself is attended with so much difficulty, that this method has fallen into disuse.

4th. That it is possible to substitute, for the local action of the leech, numerous scarifications of the external surface of the cervix uteri.

5th. That an instrument specially adapted to this organ, and rendering the operation much easier of execution, merits the attention of practitioners, and a rank in the treatment of an affection often rebellious to every other form of treatment.

